# Application and Extension of Vertical Intensity Lower-Mode in Methods for Target Depth-Resolution with a Single-Vector Sensor

**DOI:** 10.3390/s18072073

**Published:** 2018-06-28

**Authors:** Anbang Zhao, Xuejie Bi, Juan Hui, Caigao Zeng, Lin Ma

**Affiliations:** 1 Acoustic Science and Technology Laboratory, Harbin Engineering University, Harbin 150001, China; zhaoanbang@hrbeu.edu.cn (A.Z.); bixuejie@hrbeu.edu.cn (X.B.); cgzeng@hrbeu.edu.cn (C.Z.); malin@hrbeu.edu.cn (L.M.); 2 Key Laboratory of Marine Information Acquisition and Security, Harbin Engineering University, Ministry of Industry and Information Technology, Harbin 150001, China; 3 College of Underwater Acoustic Engineering, Harbin Engineering University, Harbin 150001, China; 4 National Key Laboratory of Science and Technology on Underwater Acoustic Antagonizing, China State Shipbuilding Corporation Systems Engineering Research Institute, Beijing 100036, China

**Keywords:** lower-mode correlation quantity, first three normal modes, depth resolution, improved method, Monte Carlo

## Abstract

In this paper, based on the reactive component of the vertical intensity, the method for target depth resolution has been improved. In the previous existing research results, using the reactive component of vertical intensity, the research objects for target depth resolution in shallow water, can only be the targets whose frequencies can only excite the first two normal modes, and the depth of targets whose frequencies excite more than two normal modes cannot be correctly identified. The basic idea of the improved method is to classify targets on the foundation of the lower-mode correlation quantity of the vertical intensity. Based on the improved method, we can realize depth resolution of the targets whose frequency can excite the first three normal modes so as to effectively expand the working band useful for target depth resolution. Finally, we can realize the three-dimensional target depth resolution so as to distinguish the aerial, surface and underwater targets. The feasibility of the algorithm is verified by simulation and experimental data processing.

## 1. Introduction

The acoustic field is described with two separate variables: the scalar pressure and the vectorial particle velocity variables. The pressure variable is significantly simpler to measure. Therefore, a majority of the existing acoustic applications rely on omni-directional pressure sensors. However, being a scalar variable, pressure measurements at a point in space do not provide directional information regarding the acoustic field. The particle velocity has historically been neglected, despite providing directional information regarding the acoustic field. This can be attributed to the lack of affordable sensors capable of reliable measurements [1]. However, the demand for higher performance array systems, coupled with the recent advancements in single crystal ceramic and microelectromechanical systems sensor fabrication technology, has resulted in the development of particle velocity sensors [2,3]. In general, particle velocity sensors are combined with pressure sensors in a single package to form an acoustic vector sensor (AVS). The use of signals collected by vector sensors can acquire more useful information of the target signals so as to lay a solid foundation for subsequent detection, identification, and positioning.

An acoustic vector sensor is a device that measures the three orthogonal components of the particle velocity, simultaneously with the pressure field at a single position in space. Vector sensors have been used for a long time in SONAR and target location due to their inherent spatial filtering capabilities [4]. In the early nineties, a paper by D’Spain et al. [5] received considerable attention, and during the last two decades several authors have conducted research on the signal processing theory of vector sensors ([6,7,8] and references therein). In the past decade, vector sensors have been proposed in other fields like port and waterway security [9], underwater communications [10], geoacoustic inversion [11,12,13] and geophysics [14].

The main research focus of this paper is the method for target depth resolution, which is actually a method for target category resolution. In recent years, a large number of scholars have done relevant research work in the field of target depth resolution. Bucker [15] realized the target localization based on the field information matching. Hinich [16] proposed a method for depth estimation using the maximum likelihood estimator. Shang [17] proposed an approach for target depth estimation based on the mode filtering technique. Yang adopted a method based on eigenvector decomposition technique [18] and data-based method [19] for depth estimation. Goldhahn [20] proposed a method for depth classification based on waveguide invariant adaptive matched-filtering. Matched field processing (MFP) [21,22,23,24,25,26,27,28] has also been widely used in depth estimation studies. Premus proposed a method for target depth discrimination based on matched subspace detector [29,30] and mode-filtering technology [31]. Researchers such as An [32], Premus [33] and Creamer [34] introduced a method for target depth resolution based on the modified modal scintillation index (MMSI). Mitchell [35] estimated the target depth by using power cepstrum techniques.

The above research is mainly based on the signals collected by pressure sensors, while there are still few studies on target depth resolution using the signals collected by vector sensors. Nevertheless, scholars at home and abroad have also achieved certain research results in this area.

Arunkumar and Anand [36] proposed a method for source depth estimation by matching field processing methods. Hawkes and Nehorai [37] proposed a three-dimensional localization method using distributed vector sensors. Voltz and Lu [38] proposed a method for estimating source distance and depth using the ray back propagation theory. A method for localizing acoustic sources using an array of sensors was presented by Nehorai and Paldi [39]. For an acoustic vector sensor lying in an emitter’s near-field, the methods for three-dimensional localization has been developed by Wong and Wu et al. [40,41,42,43]. Many scholars like Hui and Yu et al. [44,45,46,47,48,49] have proposed a method for target depth classification using vertical intensity signals.

In shallow water, the frequency which can excite the first two normal modes can be defined as the lower part of the Very Low Frequency (VLF: 1–100 Hz) band; and the frequency within in the higher part of VLF band can excite more than two normal modes. However, the frequency out of the VLF band is not the frequency of our research object in this paper.

The above studies on methods for target depth resolution, using signals collected by vector sensors, are mostly based on vector sensor arrays [36,37,38,39]. There are few methods for target depth resolution based on signals collected by a single vector sensor. Some of these methods offer rather high accuracies of localization and angle estimation, but have high complexity in the actual calculation [40,41,42,43]. Others are based on normal mode theory in the case of exciting only the first two normal modes [44,45,46,47,48,49], although these methods are of low complexity. When the frequency of research object can only excite the first two normal modes, the working band of the investigable object is greatly limited. That is, the depths of targets whose frequencies can excite more than two modes cannot be identified accurately and effectively, resulting in great threats to the safety and concealment of underwater platforms. Therefore, the study of target depth resolution at higher frequencies in the VLF band is very important and urgent. This paper proposes an improved method for target depth resolution according to the depth resolution requirements of target at the higher frequencies in the VLF band. The improved method can effectively solve the above problem so that the working band (in which targets can be classified correctly) has greatly expanded. Based on the proposed improved method in this paper, we can distinguish the aerial, surface and underwater targets so as to provide a solid guarantee for the safety and stability of underwater platforms.

## 2. Theory and Model

If there is no special description of the target type, all expression derivation in this section is based on the premise that the target is a harmonic source.

### 2.1. Array Manifold of the Sensing System

In this paper, the acoustic vector sensor lies in the source’s far filed and away from any reflecting boundary. The array manifold of the sensing system is [39,41]:

(1) $$a^{\left(far,no-b\right)}=\left[a_{p},a_{v_{x}},a_{v_{y}},a_{v_{z}}\right]=\left[1,\cos\theta \cos\alpha ,\sin\theta \cos\alpha ,\sin\alpha \right]$$

The above superscript “*far, no-b*” signifies that the source is in the far field and is not near any reflecting boundary [42]. The second, third and fourth component above corresponds to the velocity sensor aligned along, respectively, the *x*-axis, the *y*-axis, the *z*-axis. The first component corresponds to the pressure sensor [43]. θ is the horizontal azimuth angle (range: $0^{\circ }\sim 360^{\circ }$), the *x*-axis positive direction is $0^{\circ }$; α is the elevation angle (range: $-90^{\circ }\sim 90^{\circ }$), the horizontal plane (*xoy* plane) is $0^{\circ }$.

### 2.2. Normal-Mode Expressions of Pressure and Velocity Fields

**Figure 1:**
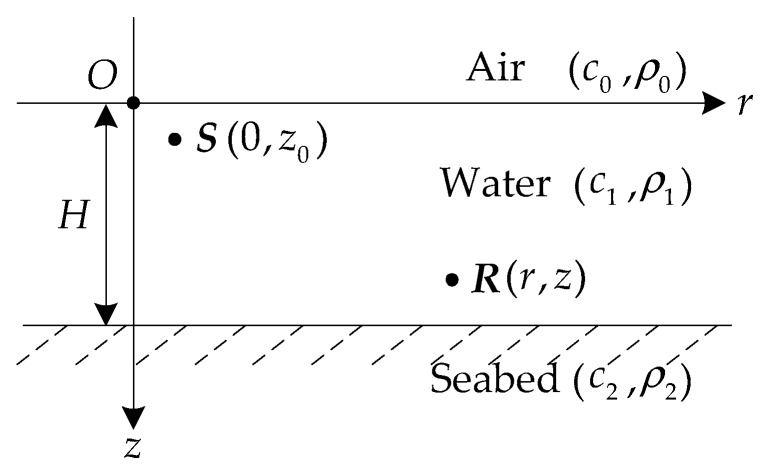
Isovelocity uniform layered media model.

The waveguide model adopted in this paper is an isovelocity uniform layered media model. The model is divided into three layers: the air, water and seabed layers, as shown in Figure 1. The sound velocity and density of each layer are constant. *H* is the sea depth. $c_{0}$, $c_{1}$, $c_{2}$ are the sound velocities in air, water, and seabed. $\rho_{0}$, $\rho_{1}$, $\rho_{2}$ are the densities of air, water and seabed layers. The sea surface is an absolutely soft interface above which the pressure is zero. The seabed is liquid half space. *O* is the origin of the coordinate system. *S* is the source position with the coordinates of (0,$z_{0}$). *R* is the receiver position with the coordinates of (*r*,*z*). $z_{0}$ is the source depth, *z* is the receiving sensor depth, and *r* is the horizontal distance between source and receiver.

In the layered media, according to the equations in [50] (p. 9), we can the expression of the potential function:

(2) $$\varphi_{N}(z_{0},z,r)=\sum_{n}\frac{2\pi j\beta_{1n}\sin(\beta_{1n}z)\sin(\beta_{1n}z_{0})}{\beta_{1n}H-\sin(\beta_{1n}H)\cos(\beta_{1n}H)-b^{2}\tan(\beta_{1n}H)\sin^{2}(\beta_{1n}H)}H_{0}^{\left(1\right)}(\xi_{n}r)$$

According to the equations in [51] (p. 58), we can get the relationships between pressure, vertical velocity fields and potential function are that:

(3) $$P(z_{0},z,r)=-j\omega \rho_{1}\cdot \varphi_{N}(z_{0},z,r)$$

(4) $$V_{z}(z_{0},z,r)=-\frac{\partial \varphi_{N}(z_{0},z,r)}{\partial z}$$

Thus, the expressions of the pressure and vertical velocity fields excited by surface or underwater targets can be expressed as follows [50,51,52,53,54]:

(5) $$P(z_{0},z,r)=2\pi \omega \rho_{1}\sum_{n}F_{n}(\xi_{n})\Psi_{n}(z_{0})\Psi_{n}(z)H_{0}^{\left(1\right)}(\xi_{n}r)=e^{-j\frac{\pi }{4}}\sqrt{\frac{8\pi }{r}}\omega \rho_{1}\sum_{n}\sqrt{\frac{1}{\xi_{n}}}F_{n}(\xi_{n})\Psi_{n}(z_{0})\Psi_{n}(z)e^{j\xi_{n}r}$$

(6) $$V_{z}(z_{0},z,r)=-2\pi j\sum_{n}F_{n}(\xi_{n})\Psi_{n}(z_{0}){\Psi^{\prime }}_{n}(z)H_{0}^{\left(1\right)}(\xi_{n}r)=-je^{-j\frac{\pi }{4}}\sqrt{\frac{8\pi }{r}}\sum_{n}\sqrt{\frac{1}{\xi_{n}}}F_{n}(\xi_{n})\Psi_{n}(z_{0}){\Psi^{\prime }}_{n}(z)e^{j\xi_{n}r}$$

(7) $$F_{n}(\xi_{n})=\frac{\beta_{1n}}{\left[x-\sin(x)\cos(x)-b^{2}\tan(x)\sin^{2}(x)\right]}$$

in which, n is the serial number of the normal mode; $\Psi_{n}(z)$ is the mode depth function; $\Psi_{n}(z)=\sin(\beta_{1n}z)$; $\beta_{in}=\sqrt{k_{i}^{2}-\xi_{n}^{2}}$; $k_{i}=\frac{\omega }{c_{i}}(i=0,1,2)$; ω is the source angular frequency. i=0,1,2 correspond to the air, water and seabed layers respectively. $\xi_{n}$ is the *n*-th order eigenvalue, and $H_{0}^{\left(1\right)}$ is the Hankel function of first kind. $x=\beta_{1n}H$, $b=\frac{\rho_{1}}{\rho_{2}}$.

### 2.3. Different Expressions of Vertical Intensity

If $A_{n}(z_{0},z,r)=\sqrt{\frac{8\pi }{\xi_{n}r}}\omega \rho_{1}F_{n}(\xi_{n})\Psi_{n}(z_{0})\Psi_{n}(z)$, $B_{n}(z_{0},z,r)=\sqrt{\frac{8\pi }{\xi_{n}r}}F_{n}(\xi_{n})\Psi_{n}(z_{0}){\Psi^{\prime }}_{n}(z)$, (5) and (6) will be changed to:

(8) $$P(z_{0},z,r)=\sum_{n}A_{n}(z_{0},z,r)e^{j(\xi_{n}r-\frac{\pi }{4})}$$

(9) $$V_{z}(z_{0},z,r)=-j\sum_{n}B_{n}(z_{0},z,r)e^{j(\xi_{n}r-\frac{\pi }{4})}$$

The vertical intensity can be expressed as [54]:

(10) $$I_{z}(r,\omega )=P(r,\omega )\cdot V_{z}^{*}(r,\omega )=I_{zA}(r,\omega )+j\cdot I_{zR}(r,\omega )$$

In the formula, the superscript * represents the complex conjugate operation. P(r,ω) and $V_{z}(r,\omega )$ are the Fourier transforms of p(r,t) and $v_{z}(r,t)$. The vertical intensity can be represented as a sum of active component $I_{zA}(r,\omega )$ and reactive component $I_{zR}(r,\omega )$. The former denotes the energy flux that can propagate to the distance, and the latter shows the energy flux that does not propagate. Substituting (8) and (9) to (10), the expression of the vertical intensity will be changed to:

(11) $$I_{z}(r,\omega )=j\cdot \left\{\sum_{n}A_{n}B_{n}^{*}+\sum_{n,n\neq m}\sum_{m}A_{n}B_{m}^{*}\left[\cos(\Delta \xi_{mn}r)+j\cdot \sin(\Delta \xi_{mn}r)\right]\right\}$$

According to (10) and (11):

(12) IzA=Re(PVz*)=−∑n,n≠m∑mAnBm∗sin(Δξmnr)

(13) IzR=Im(PVz*)=∑nAnBn∗+∑n,n≠m∑mAnBm∗cos(Δξmnr)

in which $\Delta \xi_{mn}=\xi_{m}-\xi_{n}$ is the difference between the *m*-th and *n*-th order eigenvalue.

The vertical intensity can also be expressed as the summation of the same-mode component C(r,z) and different-mode component D(r,z) [55]:

(14) $$C(r,z)=\sum_{n}A_{n}(z_{0},z,r)B_{n}^{*}(z_{0},z,r)$$

(15) $$D(r,z)=\sum_{n,n\neq m}\sum_{m}A_{n}(z_{0},z,r)B_{m}^{*}(z_{0},z,r)e^{j\Delta \xi_{mn}r}$$

(16) $$D(r,z)=D_{A}(r,z)+j\cdot D_{R}(r,z)$$

in which, $D_{A}(r,z)$ and $D_{R}(r,z)$ are the real and imaginary parts of different-mode component. According to (15) and (16), we can get that the expressions of $D_{A}(r,z)$ and $D_{R}(r,z)$ are:

(17) $$D_{A}(r,z)=\sum_{n,n\neq m}\sum_{m}A_{n}(z_{0},z,r)B_{m}^{*}(z_{0},z,r)\cos(\Delta \xi_{mn}r)$$

(18) $$D_{R}(r,z)=\sum_{n,n\neq m}\sum_{m}A_{n}(z_{0},z,r)B_{m}^{*}(z_{0},z,r)\sin(\Delta \xi_{mn}r)$$

Assuming that the source frequency can only excite the first three normal modes, the expressions of the active and reactive components of vertical intensity are:

(19) IzA=Re(PVz*)=−DR(r,z)=−[(A2B1∗−A1B2∗)sin(Δξ12r)+(A3B1∗−A1B3∗)sin(Δξ13r)+(A3B2∗−A2B3∗)sin(Δξ23r)]

(20) IzR=Im(PVz*)=C(r,z)+DA(r,z)=A1B1∗+A2B2∗+A3B3∗+(A1B2∗+A2B1∗)cos(Δξ12r)+(A1B3∗+A3B1∗)cos(Δξ13r)+(A2B3∗+A3B2∗)cos(Δξ23r)

### 2.4. Improved Method for Target Depth Resolution

**Figure 2:**
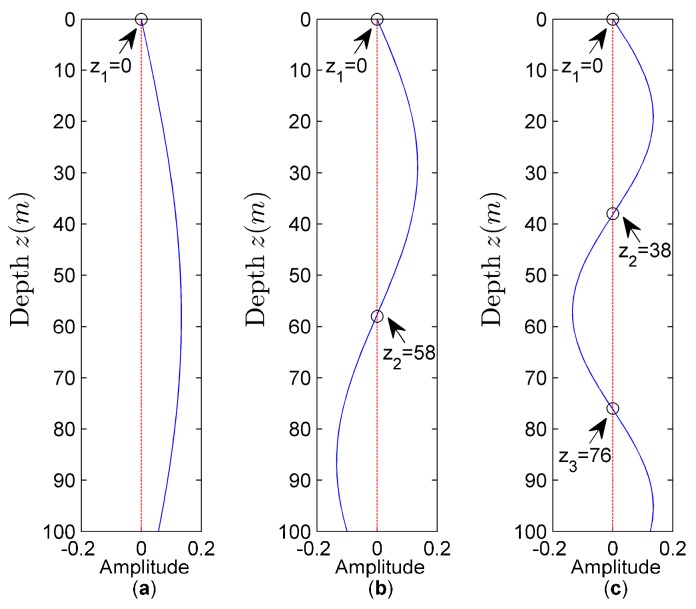
Mode depth function amplitude distribution. (**a**) First normal mode; (**b**) Second normal mode; (**c**) Third normal mode.

Using the signals collected by a vector sensor, the active and reactive components of vertical intensity can be calculated. Assuming that the waveguide environment is known, the corresponding mode depth function $\Psi_{n}(z)$ is calculated by using the expressions mentioned in Section 2.2 ($\Psi_{n}(z)=\sin(\beta_{1n}z)$; $\beta_{in}=\sqrt{k_{i}^{2}-\xi_{n}^{2}}$; $k_{i}=\frac{\omega }{c_{i}}(i=0,1,2)$), as shown in Figure 2.

From Figure 2, we can see that the *n*-th normal mode has *n* zeros, the first zero positions of each normal mode are all $z_{1}=0$. The mode function changes with depth so that its sign changes with depth. This change leads to a regular change of the vertical intensity sign with depth. Based on the mode depth function $\Psi_{n}(z)$, the values of $A_{n}$ and $B_{n}$ can be obtained for subsequent calculations.

#### 2.4.1. Harmonic Point Source

With (12)–(14), (17) and (18), we can get that:

(21) $$C(r,z)=I_{zR}-D_{A}(r,z),D_{R}(r,z)=-I_{zA}$$

knowing from (21) that:

(22) $$C(r,z)=I_{zR}+I_{zA}\cdot \frac{D_{A}(r,z)}{D_{R}(r,z)}$$

In previous research [56], it has been confirmed that when only the first two normal modes are considered, the sign distribution of $I_{zR}$ can be used for target category resolution; but when the target frequency can excite the first three normal modes, the field becomes complex so that the sign distribution of $I_{zR}$ cannot be used for target category resolution.

The improved method proposed in this paper is:

1. When the target frequency can excite first three normal modes, assuming that the field environment parameters are known.
2. $D_{A}(r,z)$ is obtained by using (17).
3. $I_{zR}$ is calculated using the pressure and vertical velocity signals collected by the sensor.
4. The same-mode component C(r,z) is extracted from $I_{zR}$ using (22).
5. The third-mode correlation quantity $C_{3}(r,z)$ is calculated based on (23).
6. $C_{3}(r,z)$ should be removed from the same-mode component C(r,z), because only the remaining lower-mode (namely, the first two normal modes) correlation quantity $C_{12}(r,z)$ is useful for later calculation.
7. Using the sign distribution of $C_{12}(r,z)$, target category resolution can be realized.

The expressions of $C_{3}(r,z)$ and $C_{12}(r,z)$ are as follows:

(23) $$C_{3}(r,z)=A_{3}B_{3}^{*}$$

(24) $$C_{12}(r,z)=C(r,z)-C_{3}(r,z)=A_{1}B_{1}^{*}+A_{2}B_{2}^{*}$$

#### 2.4.2. Radiated Noise of Surface or Underwater Targets

The expression of $C_{12}(r,z)$ given in Section 2.4.1 is for the case when the target is a harmonic point source. When the target is the noise radiated from a surface or underwater target, the improved method for target depth resolution is discussed in this section. It is assumed that the time domain waveform of the emission signal is *s*(*t*) and its frequency domain representation is S(ω). The time-domain impulse responses of the ocean waveguide are p(t) and $v_{z}(t)$. The expressions of P(ω) and $V_{z}(\omega )$ in (8) and (9) are their frequency domain representations. The time domain waveforms of the pressure and vertical velocity signals received after propagation through the waveguide are $p_{s}(t)$ and $v_{z_{s}}(t)$ in the absence of noise. While in the presence of noise, they are $p_{r}(t)$ and $q=\frac{\rho_{2}c_{2}}{\rho_{1}c_{1}}$.

$P_{s}(\omega )$ and $V_{z_{s}}(\omega )$ are the Fourier transforms of $p_{s}(t)$ and $v_{z_{s}}(t)$. In the absence of noise, the corresponding expressions are:

(25) $$p_{s}(t)=s(t)*p(t),v_{z_{s}}(t)=s(t)*v_{z}(t)$$

(26) $$P_{s}(\omega )=S(\omega )\cdot P(\omega ),V_{z_{s}}(\omega )=S(\omega )\cdot V_{z}(\omega )$$

in which, “∗” symbolizes the convolution operation.

$P_{r}(\omega )$ and $V_{z_{r}}(\omega )$ are the Fourier transforms of $p_{r}(t)$ and $q=\frac{\rho_{2}c_{2}}{\rho_{1}c_{1}}$. In the presence of noise, the expressions of the received signals in the time domain are:

(27) $$p_{r}(t)=p_{s}(t)+n_{p}(t),v_{z_{r}}(t)=v_{z_{s}}(t)+n_{v_{z}}(t)$$

in which, $n_{p}(t)$ and $n_{v_{z}}(t)$ are the corresponding time domain representations of received noise signals.

According to the equations in [53] (p. 8), the expressions of $p_{s}(t)$ and $v_{z_{s}}(t)$ are:

(28) $$p_{s}(t)=x(t)$$

(29) $$v_{z_{s}}(t)=x(t)\sin\alpha$$

in which $q=\frac{\rho_{2}c_{2}}{\rho_{1}c_{1}}$ is the target signal.

According to the equations in [53] (pp. 10–12), the time domain representation of complex acoustic intensity can be expressed as:

(30) I=p(t)⋅v(t)

Thus, the time-domain expression of the vertical intensity is:

(31) $$I_{z_{r}}(r,t)=p_{r}(t)\cdot v_{z_{r}}(t)$$

According to the Equations (27)–(31), we can get that:

(32) $$\begin{array}{cc} I_{z_{r}}(r,t) & =p_{r}(t)\cdot v_{z_{r}}(t)=\left[p_{s}(t)+n_{p}(t)\right]\cdot \left[v_{z_{s}}(t)+n_{v_{z}}(t)\right]\\ & =\;x^{2}(t)\sin\alpha +n_{p}(t)x(t)\sin\alpha +n_{v_{z}}(t)x(t)+n_{p}(t)\cdot n_{v_{z}}(t) \end{array}$$

where $n_{p}(t),n_{v_{z}}(t)$ are the isotropic noise components of pressure and vertical velocity signals received by the vector sensor which are both independent of x(t). The physical basis of complex acoustic intensity’s anti-interference performance is the correlation between pressure and velocity, whereas pressure and velocity of isotropic environment interference are irrelevant or weakly correlated. So $n_{p}(t),n_{v_{z}}(t)$ and x(t) are mutually independent. Thus in the Equation (32), only $x^{2}(t)\sin\alpha$ is the main research object, $n_{p}(t)x(t)\sin\alpha +n_{v_{z}}(t)x(t)+n_{p}(t)\cdot n_{v_{z}}(t)$ can be ignored in the later calculations. Thus the time-domain expression of the vertical intensity can be approximately expressed as:

(33) $$I_{z_{r}}(r,t)=p_{s}(t)\cdot v_{z_{s}}(t)+\Delta$$

in which, Δ is a variable with the small quantity which can be ignored.

According to (10) and (33), we can get that the approximate expression of $I_{z}(r,\omega )$ is:

(34) $$I_{z}(r,\omega )=P_{s}(\omega )V_{z_{s}}^{*}(\omega )+\Delta (\omega )$$

According to (8)–(10), (34), (12) and (13) can be changed to:

(35) IzA=Re(PVz*)=−|S(ω)|2[∑n,n≠m∑mAnBm∗sin(Δξmnr)]+Δ(ω)≈−|S(ω)|2DR(r,z)

(36) IzR=Im(PVz*)=|S(ω)|2[∑nAnBn∗+∑n,n≠m∑mAnBm∗cos(Δξmnr)]+Δ(ω)≈|S(ω)|2[C(r,z)+DA(r,z)]

Combined with (14)–(16), we can get that:

(37) $${\left|S(\omega )\right|}^{2}\approx \frac{I_{zA}}{-\sum_{n,n\neq m}\sum_{m}A_{n}B_{m}^{*}\sin(\Delta \xi_{mn}r)}$$

(38) $$C(r,z)\approx \frac{1}{{\left|S(\omega )\right|}^{2}}\left[I_{zR}+I_{zA}\cdot \frac{D_{A}(r,z)}{D_{R}(r,z)}\right]$$

Using (23), (24), (37) and (38), the numerical results of $C_{12}(r,z)$ can be obtained when the target signal is the radiated noise of a surface or underwater target. Then we can use the sign distribution of $C_{12}(r,z)$ to achieve target depth and category resolution.

#### 2.4.3. Radiated Noise of Aerial Targets

When the target signal is aerial target radiated noise, it is assumed that the time domain waveform of the emission signal is $s_{0}(t)$ and its frequency domain representation is $S_{0}(\omega )$. The time-domain impulse responses of the ocean waveguide are $p_{0}(t)$ and $v_{z_{0}}(t)$. Their frequency domain representations are $P_{0}(\omega )$ and $V_{z_{0}}(\omega )$. The time domain waveforms of the received pressure and vertical velocity signals obtained after propagation through the ocean waveguide are $p_{0r}(t)$ and $v_{z_{0r}}(t)$ respectively in the absence of noise. The corresponding frequency domain representations are $P_{0r}(\omega )$ and $V_{z_{0r}}(\omega )$. Since the aerial target can be equivalent to a surface target in the target category resolution process, the relationship between the aerial and surface target fields is as follows [56]:

(39) $$P_{0}(r,\omega )=\frac{j}{k_{0}z_{0}}P(r,\omega )$$

(40) $$V_{z0}(r,\omega )=\frac{j}{k_{0}z_{0}}V_{z}(r,\omega )$$

Combined with (34), the corresponding (35) and (36) are changed to:

(41) IzA=Re(PVz*)=|S0(ω)|2k02z02[∑n,n≠m∑mAnBm∗sin(Δξmnr)]+Δ(ω)≈|S0(ω)|2k02z02DR(r,z)

(42) IzR=Im(PVz*)=|S0(ω)|2k02z02[∑nAnBn∗+∑n,n≠m∑mAnBm∗cos(Δξmnr)]+Δ(ω)≈|S0(ω)|2k02z02[C(r,z)+DA(r,z)]

Combined with (14)–(16), we can get that:

(43) $${\left|S_{0}(\omega )\right|}^{2}\approx \frac{k_{0}^{2}z_{0}^{2}I_{zA}}{D_{R}(r,z)}$$

(44) $$C(r,z)\approx \frac{k_{0}^{2}z_{0}^{2}}{{\left|S_{0}(\omega )\right|}^{2}}\left[I_{zR}-I_{zA}\cdot \frac{D_{A}(r,z)}{D_{R}(r,z)}\right]$$

Using the expressions (23), (24), (43) and (44), the numerical results of $C_{12}(r,z)$ can be obtained when the target signal is the radiated noise of the aerial target. Then using the sign distribution of $C_{12}(r,z)$, the target depth and category resolution can be realized.

### 2.5. Method for Three-Dimensional Target Depth Resolution

**Figure 3:**
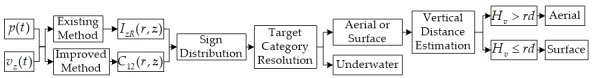
The flow chart of the target category resolution process.

Based on the existing method for target depth resolution described in previous paper [56] and the improved method proposed in this paper, combined with the method for vertical distance (between source and receiver) estimation described in [57], we can realize the three-dimensional target depth resolution so as to distinguish the aerial, surface and underwater targets. The detailed target category resolution process is shown in Figure 3.

The concrete steps of the method for three-dimensional target depth (category) resolution are the following:

1. Using the collected signals p(t) and $v_{z}(t)$, based on the existing and improved methods for target depth resolution, the sign distribution of $I_{zR}(r,z)$ and $C_{12}(r,z)$ can be obtained.
2. Using the sign distribution, the target category resolution can be realized so as to distinguish between the aerial or surface targets and underwater targets.
3. Based on the method for vertical distance estimation, we can get the vertical distance between the source and receiver which is defined as $H_{v}$.
4. Through comparing the value of vertical distance $H_{v}$ with the sensor depth *rd*, we can distinguish the aerial and surface targets: if $H_{v}>rd$, the target can be identified as an aerial target; if $H_{v}\leq rd$, the target can be identified as a surface target.
5. Finally, we can distinguish the aerial, surface and underwater targets so that the three-dimensional target category (depth) resolution has been realized.

## 3. Simulation Data and Results

All the marine environmental parameters of the simulations in this section are the same as shown in Table 1. However, the target simulation parameters in each subsection of this section are different, as shown later. The pressure and vertical velocity signals are all collected by a single three-dimensional vector sensor in the following simulations.

### 3.1. Harmonic Point Source

**Figure 4:**
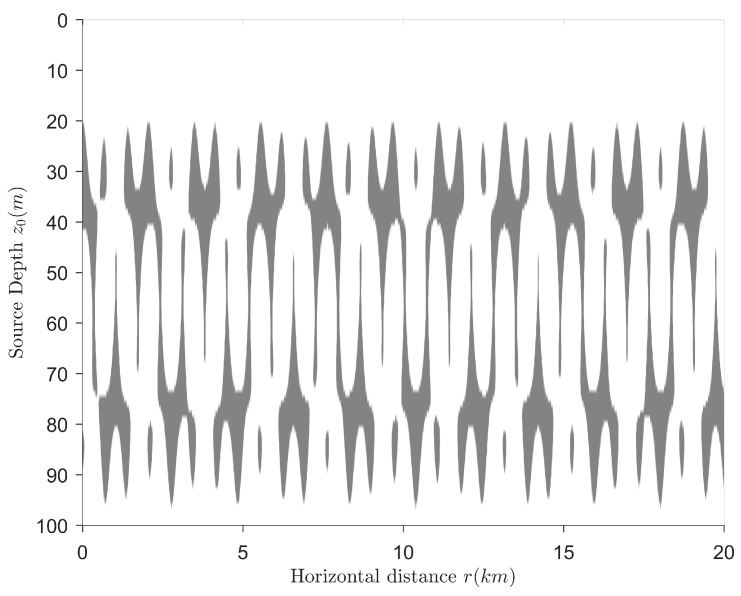
The sign distribution of $I_{zR}(r,z)$.

**Figure 5:**
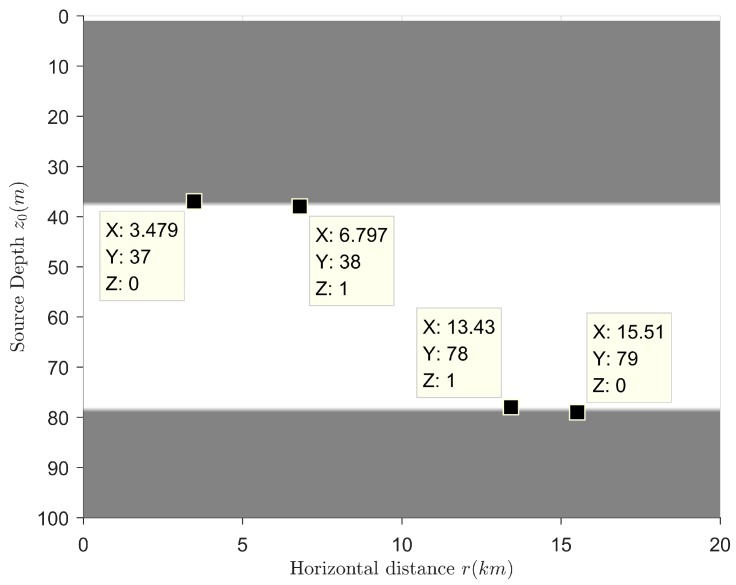
The sign distribution of $C_{12}(r,z)$.

The target is a point source that radiates single-frequency harmonic wave whose frequency is f=80 Hz. The receiving sensor depth is 50 m. The range of source depth is 1~100 m. The range of horizontal distance is 1~20 km. The sign distributions of $I_{zR}(r,z)$ and $C_{12}(r,z)$ are shown in Figure 4 and Figure 5, respectively. Gray means that the sign is negative and the sign value is defined as 0. White means that the sign is positive and the sign value is defined as 1.

Figure 4 has proved that when the source frequency can excite the first three normal modes, the sign distribution of $I_{zR}(r,z)$ becomes very complicated so that it cannot be used for target depth resolution. As can be seen from Figure 5, the sign distribution of $C_{12}(r,z)$ has a critical depth of sign, and this critical depth is about 37 m. When the source depth is less than this depth, the sign of $C_{12}(r,z)$ is negative, and the source located at this position can be identified as a surface target. When the source depth is greater than this depth, the sign of $C_{12}(r,z)$ is positive, and the source at this place can be identified as an underwater target. Therefore, under the assumption that there are no targets sailing near the seabed, the source depth resolution can be realized by using the sign distribution of $C_{12}(r,z)$. The source category can be identified at the same time. The frequency range (in which the target category can be identified) is effectively expanded.

### 3.2. Relationship between Target Category-Resolution Accuracy and SNR

The principle of motion parameter selection is:

1. The research object of this paper is moving target.
2. Select the simplest motion model: uniform linear motion model.
3. If the sailing time of the target is long enough, the target will move away from the receiver finally. Thus there are two different considerable motion tendencies: (1) move away from the receiver after getting close to it; (2) move away from the receiver all the time.
4. The considerable target types are surface and underwater, because the aerial targets are equivalent to surface targets during the three-dimensional target category resolution.
5. Based on rich references, the target velocities are all supposed to be lower than 15 m/s.
6. The platform is generally stationary or sailing in the low velocity, so the value of the platform velocity is set to 2 m/s.

In Section 3.2, the motion parameters are that: the surface target moves away from the receiver after getting close to it; the underwater target moves away from the receiver all the time. The other detailed target simulation parameters are shown in Table 2 and Table 3. *SNR* is the abbreviation of Signal-Noise Ratio.

**Figure 6:**
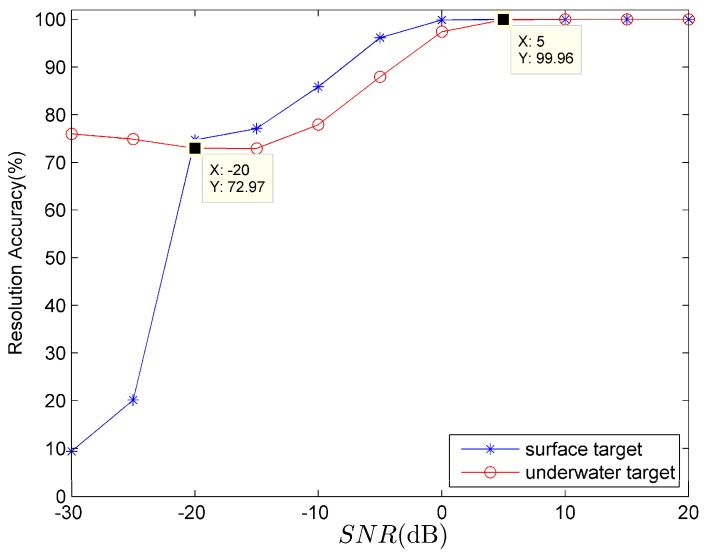
The relationship between target category-resolution accuracy and *SNR*.

When the target line spectrum frequency can excite the first three normal modes, the target category-resolution accuracies of surface (line marked with star symbol) and underwater (line marked with circle symbol) targets using the 20th second data are shown in Figure 6.

The frequencies of surface and underwater targets are *f* = 70 Hz and *f* = 80 Hz respectively. It can be seen from Figure 6 that: when *SNR* > −20 dB, whether the target type is surface or underwater, their target resolution accuracies are both greater than 70%; when SNR≥ 5 dB, the target resolution accuracies both approach 100%.

### 3.3. Target Depth Resolution Results

In Section 3.3, the motion parameters are that: the surface target moves away from the receiver after getting close to it; the underwater target moves away from the receiver all the time. The other detailed target simulation parameters are shown in Table 4 and Table 5.

**Figure 7:**
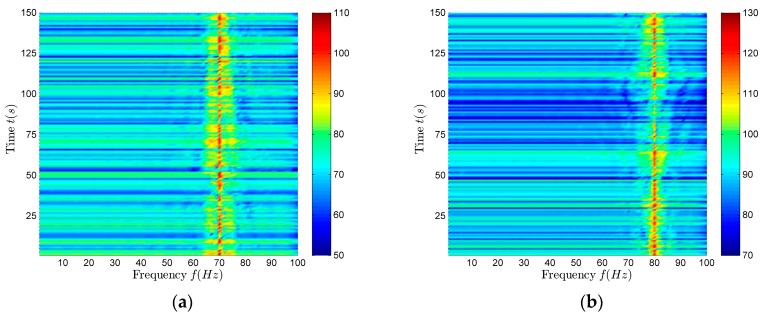
The time-frequency distribution of surface and underwater targets. (**a**) Surface target; (**b**) Underwater target.

The corresponding time–frequency distributions of surface and underwater targets are shown in Figure 7a,b. From Figure 7a,b, the target line spectrum frequency can be estimated. The target depth resolution can be conducted at the corresponding frequency. The resolution results are shown in Table 6. According to the data in Table 6, it can be seen that in the shallow water, using the method proposed in this paper, the depth resolution of the targets (at the higher frequencies in the VLF band) can be achieved stably and accurately.

## 4. Sea Experiment Data and Results

**Figure 8:**
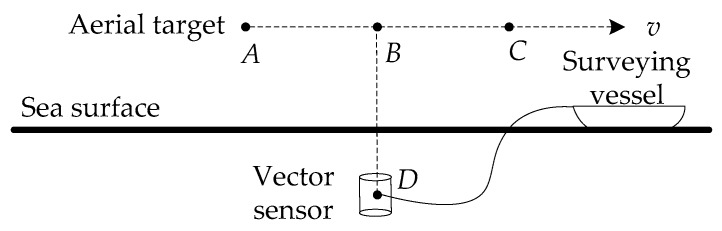
The profile of the sea experiment layout.

The sea experiment used a piezoelectric ceramic vector sensor, namely an accelerometer, to acquire aerial target radiated noise signal. The experiment layout is shown in Figure 8. The three-dimensional low-frequency vector sensor is suspended underwater. Based on the collected pressure and velocity signals, the sign distribution of the lower-mode correlation quantity $C_{12}(r,z)$ is used to achieve the depth resolution of the target (at the higher frequencies in the VLF working band) so as to realize the target category resolution. The improved method proposed in this paper is mainly aimed at the target line spectrums whose frequencies can excite the first three normal modes.

**Figure 9:**
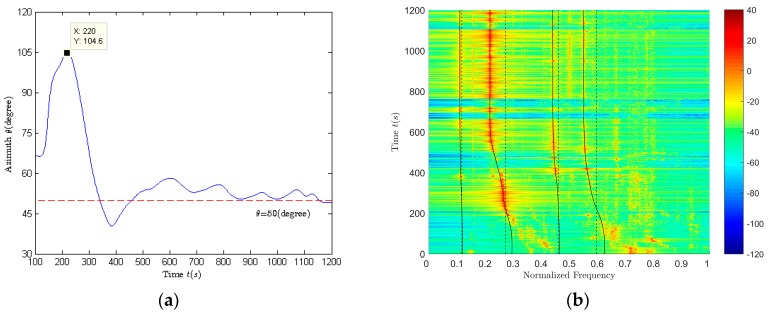
The azimuth and frequency estimation results. (**a**) Azimuth estimation results; (**b**) The normalized spectrum of $v_{z}$ signal and its frequency sequence extraction results.

The experiment condition is that the sea depth is 50 m, the receiver depth is 25 m, and the target velocity is 80 km/h. The target appeared roughly after 220 s, at some point in the vicinity of the top of the sensor, and then away. The vector sensor was at point *D*. The aerial target ran from point *A* to point *C*, and flied at a constant velocity *v*. After the aerial target was in place, it flew over the vector sensor mounted on the underwater platform and then went away [56,57]. After the sea experiment data is processed, the azimuth and frequency estimation are carried out. The azimuth and frequency estimation results are shown in Figure 9. Based on the above estimation results, we can get the target parameter estimation results as listed in Table 7. The detailed estimation method is shown in [57].

Among them, ${\hat{f}}_{01}$ is the base frequency of the aerial target; $\hat{\psi }$ is the heading angle; $\hat{v}$ is the target velocity; $\hat{p}$ is the closest distance between source and receiver in horizontal direction; $H_{v}$ is the distance between source and receiver in vertical direction.

**Figure 10:**
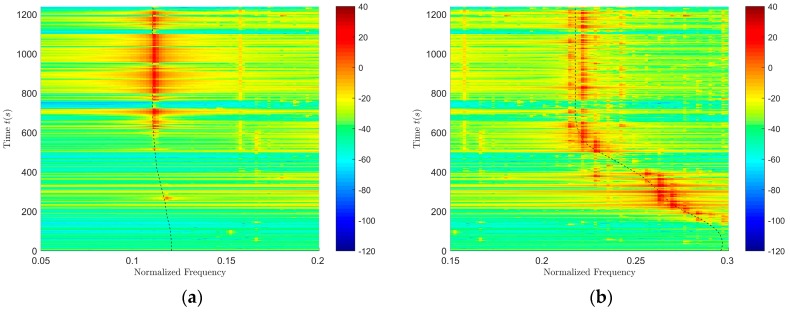
The normalized spectrum of $v_{z}$ signal in the band where the first two line spectrums locate and its frequency sequence extraction results. (**a**) The first line spectrum and its frequency sequence extraction results; (**b**) The second line spectrum and its frequency sequence extraction results.

Figure 10 shows the time-frequency distribution in the frequency band where the first two line spectrums locate and the corresponding frequency sequence extraction results (dotted lines in black) of the first two line spectrums respectively. Figure 10a corresponds to the band where the first line spectrum locates. Figure 10b corresponds to the band where the second line spectrum locates. The source frequency estimation results of the first two line spectrums are 0.11 and 0.2167. The reference value is the maximum frequency of the working band selected in Figure 9b. Based on the frequency estimation results obtained in [56,57] and the horizontal distance compensation results in [57], the depth resolution of aerial targets can be achieved using (39)–(44). In [56], the method using the line spectrum whose frequency only excites the first two normal modes to perform target depth resolution (abbreviated as method 1) is described in detail and the results are given. The improved method proposed in this paper (abbreviated as method 2) can provide the target depth resolution results using spectrum whose frequency can excite the first three normal modes, and the method in [56] cannot use the target line spectrum whose frequency can excite the first three normal modes to perform the depth resolution.

The data in Table 8 are the target category resolution accuracies based on the existing method of [56] and the improved method proposed in the paper. $T_{i}$ indicates the length of integration time. From the data in Table 8, it can be seen that resolution accuracy is low or even incorrect by processing the line spectrum 2 based on the method 1. It means that we cannot use method 1 to process the second line spectrum for target category resolution because its application environment is limited. We can only use method 1 to process the spectrum whose frequency can only excite the first two normal modes, while the frequency of the second line spectrum can excite the first three normal modes. Using the method 2 proposed in this paper, the target category can be correctly identified by processing the second line spectrum. The appropriate integration time length can be selected to effectively improve the target category resolution accuracy.

Through the above target category resolution results in Table 8, the target category can first be identified as an aerial or a surface target correctly. Then through the comparison of vertical distance estimation result $H_{v}=\;150\;m$ shown in Table 7 with the known sensor depth *rd* = 25 m, we can get that: $H_{v}>rd$. Then according to the method for three-dimensional target depth (category) resolution described in Section 2.5, we can get that: $H_{v}>rd$ means that the target can be identified as an aerial target when the target has already been identified as an aerial or a surface target. In conclusion, after obtaining the pressure and velocity signals, through the above methods, we can realize the three-dimensional depth resolution so as to distinguish the aerial, surface and underwater targets.

## 5. Conclusions

In this paper, through the theoretical study of the different expressions of vertical intensity, the reason why the reactive component of vertical intensity can only be used for depth resolution of the targets whose frequency can excite the first two normal modes is studied. It is actually the reason why the field interference structure at frequency which can excite more than two normal modes is more complex. Both of the reasons mentioned above are that there are more zeros of normal modes when the higher normal modes (more than two normal modes) has been excited, resulting in a more complicated field interference structure, so that the depth resolution can not be conducted by using the reactive component of vertical intensity purely. In this paper, under the assumption that the field environment information is known, the field information simulation can be performed. The corresponding calculations are used to eliminate the higher-mode correlation quantity of the same-mode component of vertical intensity and keep the lower-mode correlation quantity only. Using the lower-mode correlation quantity of the same-mode component of vertical intensity at frequencies which can excite more than two normal modes, target depth resolution can be performed. Then the bandwidth of the target working band useful for target depth resolution has been expanded effectively, so the safety and concealment of the underwater platform have also been improved availably. Monte Carlo simulations verify the feasibility and accuracy of the improved algorithm proposed in this paper for surface or underwater target’s depth resolution. Since the aerial target can be equivalent to a surface target in target depth resolution, this paper also presents a calculation method for aerial target depth resolution using the improved algorithm. The feasibility and stability of the proposed algorithm have been validated through sea experiment data processing.

## Figures and Tables

**Table 1:** The marine environmental simulation parameters.

Parameters	Value	Parameters	Value
Sea depth *H*	100 m	Sound velocity in the air $c_{0}$	334 m/s
Water density $\rho_{1}$	1026 kg/m^3^	Sound velocity in the water $c_{1}$	1480 m/s
Seabed density $\rho_{2}$	1769 kg/m^3^	Sound velocity in the seabed $c_{2}$	1550 m/s

**Table 2:** Target simulation parameters of the surface target.

Parameters	Value	Parameters	Value	Parameters	Value
Target type	surface	Platform velocity	2 m/s	Tonnage	10,000 t
Target depth	5 m	Closest distance	5800 m	Range of *SNR*	−30~20 dB
Heading angle	$50^{\circ }$	Initial distance	7200 m	Sensor depth	50 m
Target velocity	9 m/s	Target frequency	70 Hz	Sailing time	150 s

**Table 3:** Target simulation parameters of the underwater target.

Parameters	Value	Parameters	Value	Parameters	Value
Target type	underwater	Platform velocity	2 m/s	Tonnage	10,000 t
Target depth	60 m	Closest distance	8300 m	Range of *SNR*	−30~20 dB
Heading angle	$20^{\circ }$	Initial distance	9600 m	Sensor depth	50 m
Target velocity	10 m/s	Target frequency	80 Hz	Sailing time	150 s

**Table 4:** Target simulation parameters of the surface target.

Parameters	Value	Parameters	Value	Parameters	Value
Target type	surface	Platform velocity	2 m/s	Tonnage	10,000 t
Target depth	5 m	Closest distance	5800 m	*SNR*	0 dB
Heading angle	$50^{\circ }$	Initial distance	7200 m	Sensor depth	50 m
Target velocity	9 m/s	Target frequency	70 Hz	Sailing time	150 s

**Table 5:** Target simulation parameters of the underwater target.

Parameters	Value	Parameters	Value	Parameters	Value
Target type	underwater	Platform velocity	2 m/s	Tonnage	10,000 t
Target depth	60 m	Closest distance	8300 m	*SNR*	0 dB
Heading angle	$20^{\circ }$	Initial distance	9600 m	Sensor depth	50 m
Target velocity	10 m/s	Target frequency	80 Hz	Sailing time	150 s

**Table 6:** Target category-resolution accuracy.

Target Type	Line Spectrum Frequency	Accuracy
Surface	70 Hz	92.2537%
Underwater	80 Hz	88.2388%

**Table 7:** The parameter estimation results.

Motion Parameters	Estimation Results
Source frequency ${\hat{f}}_{01}$	0.1167
Heading angle $\hat{\psi }$	53.0°
Velocity $\hat{v}$	18.8 m/s
Closest distance $\hat{p}$	744 m
Vertical distance $H_{v}$	150 m

**Table 8:** Target category-resolution accuracy (830–1200 s).

Research Object	Line Spectrum 1 (Method 1)	Line Spectrum 2 (Method 1)	Line Spectrum 2 (Method 2)
Resolution accuracy ($T_{i}=4\;s$)	67.9245%	43.9353%	70.8895%
Resolution accuracy ($T_{i}=6\;s$)	72.0430%	53.7634%	61.5591%
Resolution accuracy ($T_{i}=8\;s$)	72.1925%	53.7433%	57.4866%
Resolution accuracy ($T_{i}=10\;s$)	70.9333%	53.0667%	86.6667%
